# Influence of Vitamin E Intake on Sexual Function and Depressive Symptoms in Young Women with Euthyroid Autoimmune Thyroiditis Undergoing Vitamin D Therapy: A Pilot Study

**DOI:** 10.3390/ijms27156713

**Published:** 2026-07-27

**Authors:** Robert Krysiak, Karolina Kowalcze, Johannes Ott, Giovanni Cangelosi, Simona Zaami, Bogusław Okopień

**Affiliations:** 1 Department of Internal Medicine and Clinical Pharmacology, Medical University of Silesia, Medyków 18, 40-752 Katowice, Poland; bokopien@sum.edu.pl; 2 Department of Pediatrics in Bytom, Faculty of Health Sciences in Katowice, Medical University of Silesia, Stefana Batorego 15, 41-902 Bytom, Poland; kkowalcze@sum.edu.pl; 3 Department of Pathophysiology, Faculty of Medicine, Academy of Silesia, Rolna 43, 40-555 Katowice, Poland; 4 Clinical Division of Gynecologic Endocrinology and Reproductive Medicine, Department of Obstetrics and Gynecology, Medical University of Vienna, 1090 Vienna, Austria; johannes.ott@meduniwien.ac.at; 5 School of Pharmacy, Experimental Medicine and “Stefania Scuri” Public Health Department, University of Camerino, 62032 Camerino, Italy; giovanni01.cangelosi@unicam.it; 6 Department of Anatomical, Histological, Forensic and Orthopedic Sciences, Sapienza University of Rome, 00161 Rome, Italy; simona.zaami@uniroma1.it

**Keywords:** depressive symptoms, fat-soluble vitamins, sexual dysfunction, hypothalamic-pituitary-thyroid axis, thyroid autoimmunity, women’s health

## Abstract

Euthyroid autoimmune thyroiditis (Hashimoto’s disease) has been shown to negatively affect female sexual health; however, this adverse impact appears to be mitigated by vitamin D therapy. Emerging research suggests that vitamin E may reduce susceptibility to autoimmune thyroiditis and that supplementation could contribute to improved sexual health outcomes. The present study aimed to investigate whether vitamin E status influences the effects of exogenous vitamin D on female sexual function and depressive symptoms in individuals with this condition. This pilot study included three cohorts of young women with Hashimoto’s disease, matched for age, thyroid antibody titers, and 25-hydroxyvitamin D levels, all exhibiting normal TSH and free thyroid hormone concentrations. The cohorts differed in vitamin E intake: below the recommended daily allowance (group 1), adequate intake (group 2), and high intake (exceeding 400 IU daily; group 3). All participants received vitamin D at a daily dose of 100 µg for six months. Serum hormone levels, thyroid antibody titers, and calculated indices of thyroid homeostasis were evaluated at enrollment and at the conclusion of the study. Female sexual function and depressive symptoms were also evaluated at both time points using validated instruments: the Female Sexual Function Index (FSFI) and the Beck Depression Inventory-II (BDI-II). At enrollment, group 2 scored higher than the other cohorts in the sexual desire and arousal domains. Vitamin D supplementation increased serum 25-hydroxyvitamin D in all groups. The decrease in antibody titers was most pronounced in group 2, and only in this group did vitamin D enhance thyroid secretory capacity and increase testosterone levels. In group 2, treatment led to improvements across all FSFI domains and the total score. In group 1, positive effects were limited to the lubrication and lack of pain/discomfort domains, whereas group 3 showed no changes in sexual function. Improvements in female sexual function correlated with vitamin E intake, reductions in antibody titers, and increases in testosterone levels. Significant improvements in depressive symptoms, as measured by the BDI-II, were observed exclusively in group 2. Adequate vitamin E intake is essential to achieve the full effects of vitamin D on female sexual function and mood in young euthyroid patients with Hashimoto’s disease.

## 1. Introduction

Autoimmune thyroiditis, commonly referred to as Hashimoto’s disease, is recognized as one of the most prevalent human disorders, the most frequent organ-specific autoimmune condition, and the primary cause of hypothyroidism (excluding regions affected by iodine deficiency) [1,2]. The disease is characterized by antibody-mediated cytotoxicity and programmed cell death, leading to the gradual destruction of thyroid follicular cells. Consequently, normal thyroid parenchyma is progressively replaced by lymphocytic infiltration and fibrotic tissue deposition [3]. One of the less well-recognized complications of autoimmune thyroiditis is sexual dysfunction, which is more frequently observed in women, who represent the majority of affected patients. Although more pronounced in the presence of hypothyroidism, sexual dysfunction may already occur in the euthyroid stage [4,5]. In such cases, it may be associated with increased production of pro-inflammatory mediators, subtle alterations in thyroid function, inadequate micronutrient intake, and/or reduced testosterone levels [6]. However, the possibility of confounding effects of nonspecific factors, including dysmenorrhea, smoking, stress, and traumatic life experiences, in these studies cannot be completely excluded.

Autoimmune thyroiditis development and progression have been linked recently to altered vitamin D (calciferol) homeostasis. Patients with autoimmune thyroid disease exhibit a higher prevalence of vitamin D deficiency than healthy individuals or those with other endocrine disorders [7] and display lower mean 25-hydroxyvitamin D (25OHD) levels [8]. Vitamin D deficiency severity correlates directly with thyroid peroxidase antibody (TPOAb) titers, Hashimoto’s thyroiditis duration, and thyroid gland volume [9]. Supplementation significantly reduces TPOAb and thyroglobulin antibody (TgAb) titers, an effect that intensifies over time [10,11]. The beneficial effect of vitamin D is likely mediated by regulated dendritic cell maturation and inhibited antigen presentation, which curtail self-reactive T-cell activation [12]. Consequently, adequate vitamin D levels promote immune tolerance, directly controlling thyroid autoimmunity [13].

Vitamin D deficiency appears to be associated with sexual dysfunction, with the risk increasing as the degree of deficiency increases [14,15]. The beneficial effects of exogenous vitamin D on sexual function in women of reproductive age suggest that this dysfunction may be reversible. Improvements were reported both in women with isolated vitamin D deficiency and in those with vitamin D deficiency coexisting with polyendocrine metabolic ovarian syndrome following administration of high doses of this vitamin (an average of 50,000 IU per week) [16,17,18]. In patients with autoimmune thyroiditis, the beneficial effects of moderate doses of vitamin D (4000 IU daily) were observed regardless of baseline vitamin D status and were more pronounced than those associated with other management options for this condition, namely selenomethionine and myo-inositol [6]. However, it remains unknown whether these effects are modified by other modifiable lifestyle factors.

The term vitamin E refers to a group of eight structurally related, fat-soluble compounds capable of neutralizing free radicals and reducing oxidative stress [19]. Owing to its potent antioxidant and immunomodulatory properties, vitamin E may play a role in preventing the development and progression of various autoimmune diseases [20,21]. To date, three studies have evaluated the relationship between dietary vitamin E intake and autoimmune thyroiditis. Low vitamin E intake in men was associated with an increased risk of Hashimoto’s disease compared with moderate and high intake levels [22]. Moreover, TPOAb and TgAb titers in patients with autoimmune thyroiditis showed an inverse correlation with vitamin E intake [23]. However, no association between vitamin E intake and the risk of developing Hashimoto’s disease was demonstrated in the third study, possibly due to its significant methodological limitations [24]. One proposed mediator of the putative biological link between vitamin E and Hashimoto’s disease is carboxyethylhydroxychromanic acid (CEHC), the final metabolite of vitamin E catabolism, which has been reported to interact functionally with retinoid X receptors (RXRs) [25].

Recent studies indicate that reducing oxidative stress may benefit female sexual function. In women with diabetes, lower arylesterase activity—a key antioxidant enzyme involved in preventing lipoprotein oxidation and serving as a marker of oxidative stress—was associated with sexual dysfunction [26]. Supplementation with 100 IU/day of vitamin E combined with ginseng improved libido and sexual satisfaction in young women [27]. Similarly, in premenopausal women, vitamin E supplementation (50 mg/day) improved sexual function, with greater benefits observed when administered concomitantly with another antioxidant compound, saffron [28]. Finally, in postmenopausal women, 100 IU/day of vitamin E improved desire, arousal, lubrication, and orgasm to a degree comparable to that achieved with intravaginal estrogen therapy [29].

Collectively, available evidence suggests that vitamin D and vitamin E may have beneficial effects on thyroid autoimmunity and female sexual response. However, their potential interplay has not been investigated. Therefore, the objective of the present study was to evaluate the effect of vitamin D supplementation on sexual function in euthyroid women with autoimmune thyroiditis while accounting for vitamin E intake and exploring whether the observed effects were associated with mood-related outcomes.

## 2. Results

### 2.1. Baseline Demographic, Anthropometric, and Laboratory Parameters of the Study Cohorts

At study entry, no significant differences were identified among the patient groups with regard to age, educational attainment, occupational status and type of work performed, physical activity, number of sexual partners, number and duration of marriages, number of deliveries and miscarriages, smoking status, body mass index, arterial blood pressure, daily dietary vitamin D intake, or the prevalence of vitamin D deficiency or insufficiency. In addition, the groups did not differ in TPOAb and TgAb titers, serum TSH concentrations, free thyroid hormone levels, estradiol and prolactin concentrations, or the values of the Jostel index, SPINA-GT, SPINA-GD, Female Sexual Function Index (FSFI) score, and Beck Depression Inventory—Second Edition (BDI-II) score. The only statistically significant differences observed were related to vitamin E intake, the percentage of vitamin E supplement users, and testosterone concentrations. Vitamin E intake and the percentage of vitamin E supplement users were highest in group 3 and lowest in group 1, whereas testosterone concentrations were higher in group 2 than in the other groups (Table 1).

### 2.2. Flow of Participants Through the Study

**Figure 1:**
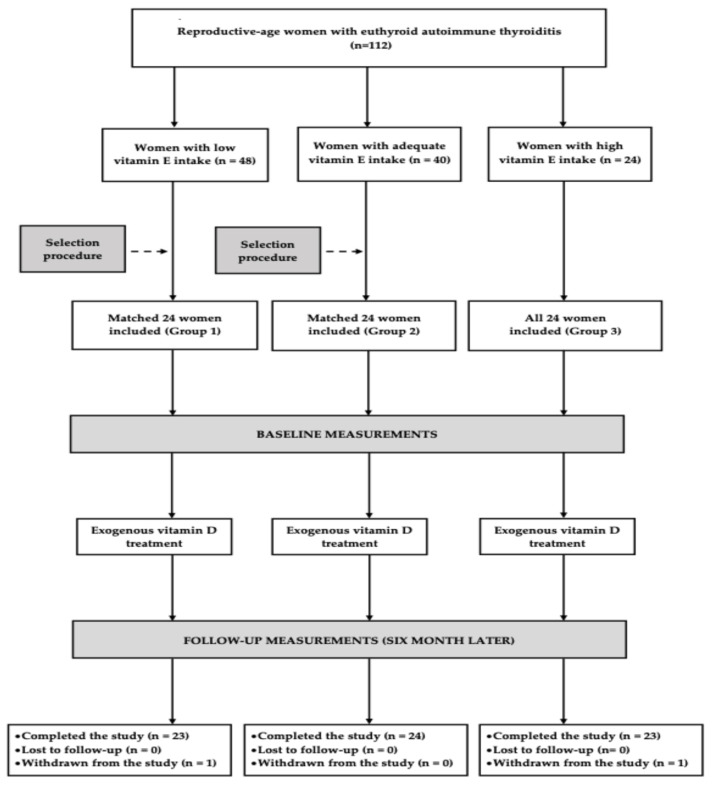
Flow of participants through the study.

Exogenous vitamin D treatment was well tolerated, and no treatment-related adverse effects were reported. A total of 70 participants (23 from group 1, 24 from group 2, and 23 from group 3) completed the study, representing 97% of those enrolled. Two patients, one from group 1 and one from group 3, withdrew due to non-compliance with the prescribed treatment (Figure 1). Given the observed effect size, final sample size, and a predefined α level, a post hoc power analysis indicated that the study achieved 81% statistical power to detect the observed effect and reject the null hypothesis under the specified assumptions.

The mean vitamin D intake (with food and supplements) during the study was 115 ± 10 µg in group 1, 112 ± 10 µg in group 2, and 116 ± 11 µg in group 3, with no significant differences between the groups. However, in each group, this intake was higher than the baseline intake (*p* < 0.001). At the end of the study, no cases of vitamin D deficiency were observed in any of the groups. Meanwhile, vitamin E intake was 8 ± 4 mg, 40 ± 53 mg, and 835 ± 228 mg in groups 1, 2, and 3, respectively, with the highest intake observed in group 3 and the lowest in group 1. However, in all groups, these values did not differ from those recorded before the study began. No significant between-group differences were observed throughout the study in energy intake or in the intake of protein, fat, or carbohydrates. Similarly, no differences in physical activity levels were observed between the groups.

### 2.3. Biochemical Variables

**Figure 2:**
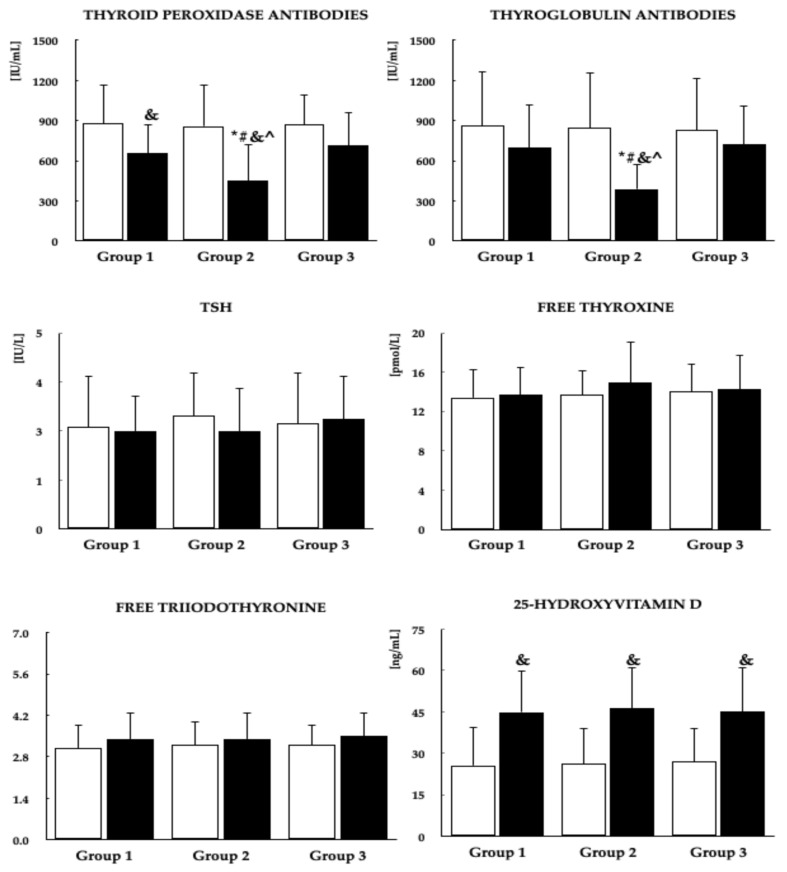
Effect of vitamin D on thyroid antibody titers, hypothalamic–pituitary–thyroid axis activity, and 25-hydroxyvitamin D levels in reproductive-age women with euthyroid autoimmune thyroiditis, stratified by vitamin E status. Values are presented as means with standard deviations. Group 1 had low vitamin E intake, group 2 had adequate vitamin E intake, and group 3 had high vitamin E intake. White bars represent measurements at study entry, and black bars represent measurements at the end of the study. Statistical analysis: ANCOVA with Bonferroni correction for between-group comparisons; paired *t* tests for within-group comparisons. * *p* < 0.05 compared with the corresponding values in group 1. ^#^ *p* < 0.05 compared with the corresponding values in group 3. ^&^ *p* < 0.05 compared with baseline values within the same group. ^^^ *p* < 0.05 indicates that percentage changes from baseline were greater than those observed in the other groups.

**Figure 3:**
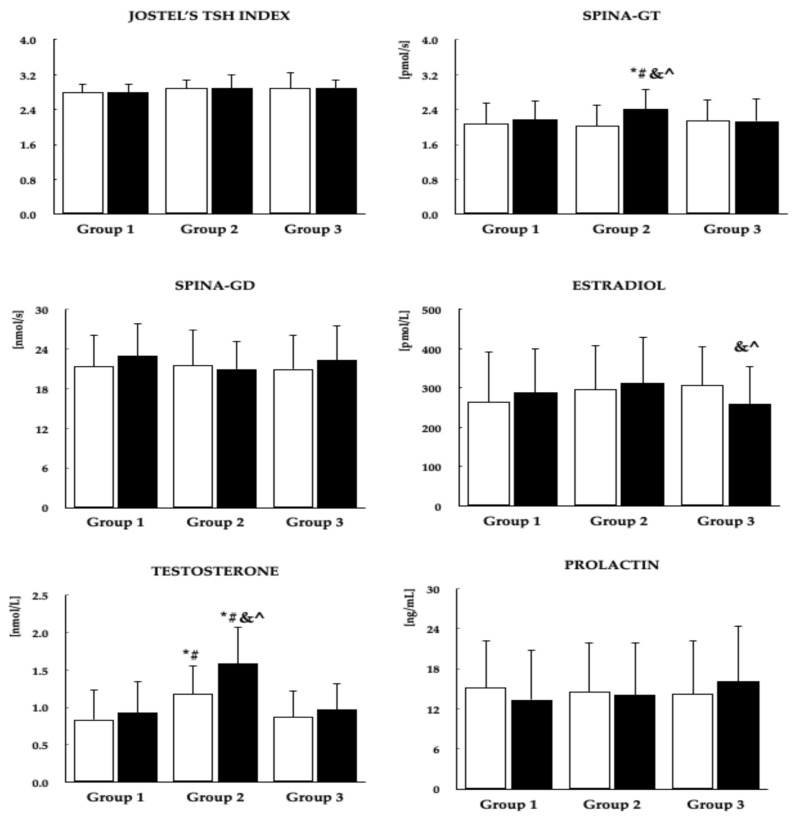
Effect of vitamin D on calculated parameters of thyroid homeostasis and the remaining hormones in reproductive-age women with euthyroid autoimmune thyroiditis, stratified by vitamin E status. Values are presented as means with standard deviations. Group 1 had low vitamin E intake, group 2 had adequate vitamin E intake, and group 3 had high vitamin E intake. White bars represent measurements at study entry, and black bars represent measurements at the end of the study. Statistical analysis: ANCOVA with Bonferroni correction for between-group comparisons; paired *t* tests for within-group comparisons. * *p* < 0.05 compared with the corresponding values in group 1. ^#^ *p* < 0.05 compared with the corresponding values in group 3. ^&^ *p* < 0.05 compared with baseline values within the same group. ^^^ *p* < 0.05 indicates that percentage changes from baseline were greater than those observed in the other groups.

In groups 1 and 2, exogenous vitamin D reduced TPOAb titers, whereas its effect on TgAb titers and SPINA-GT was observed only in group 2. An increase in 25OHD concentrations was noted in all study groups. Vitamin D caused a decrease in estradiol levels only in group 3. No changes were observed in any study group regarding TSH, free thyroxine, free triiodothyronine, or prolactin levels, nor was there any impact of vitamin D supplementation on Jostel’s TSH index or SPINA-GD values. The percentage changes in the effect of vitamin D on TPOAb, TgAb, SPINA-GT, and testosterone were more pronounced in group 2 than in the other groups, whereas changes in estradiol differed between group 3 and groups 1 and 2. On the final day of the study, differences were observed between group 2 and groups 1 and 3 in TPOAb, TgAb, SPINA-GT, and testosterone levels (Figure 2 and Figure 3).

### 2.4. Sexual Functioning

**Figure 4:**
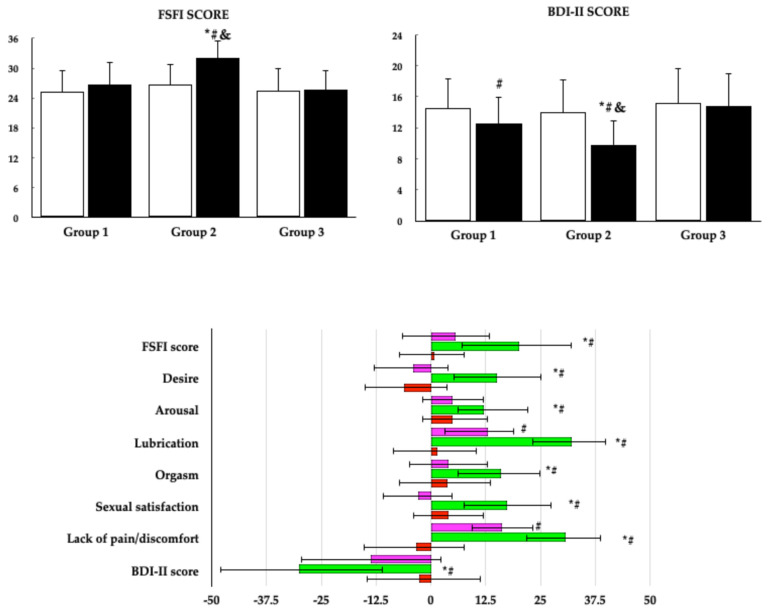
Effect of vitamin D on total FSFI and BDI-II scores and percentage changes from baseline in sexual functioning and depressive symptoms in reproductive-age women with euthyroid autoimmune thyroiditis, stratified by vitamin E status. Values are presented as means with standard deviations. Group 1 had low vitamin E intake, group 2 had adequate vitamin E intake, and group 3 had high vitamin E intake. White bars represent measurements at study entry, and black bars represent measurements at the end of the study. Statistical analysis: ANCOVA with Bonferroni correction for between-group comparisons; paired *t* tests for within-group comparisons. * *p* < 0.05 compared with the corresponding values in group 1. ^#^ *p* < 0.05 compared with the corresponding values in group 3. ^&^ *p* < 0.05 compared with baseline values within the same group.

**Figure 5:**
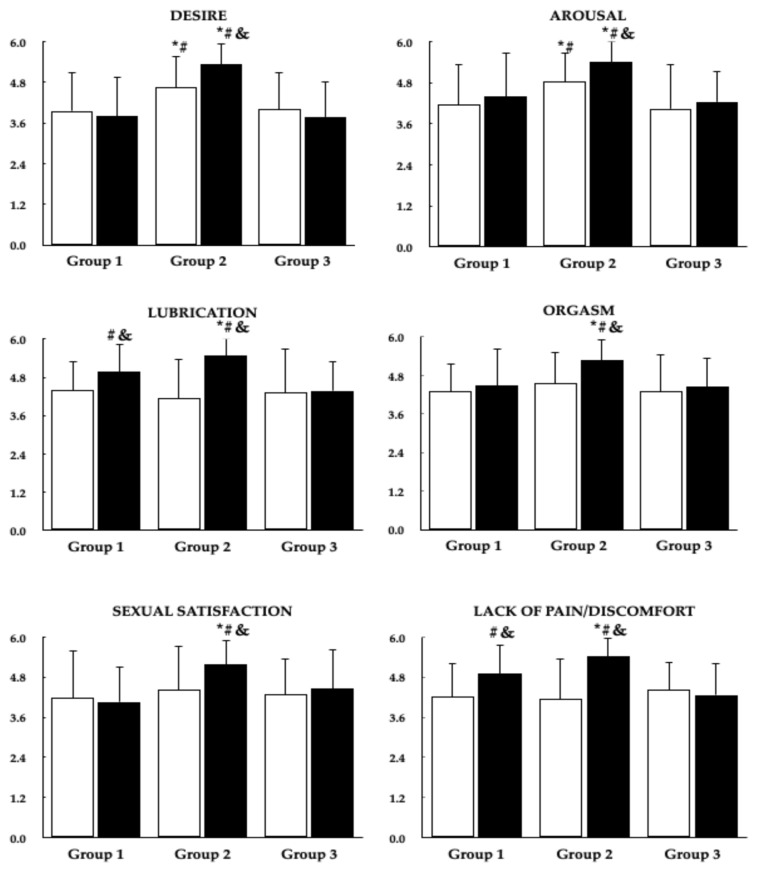
Effect of vitamin D on components of sexual function in reproductive-age women with euthyroid autoimmune thyroiditis, stratified by vitamin E status. Values are presented as means with standard deviations. Group 1 had low vitamin E intake, group 2 had adequate vitamin E intake, and group 3 had high vitamin E intake. White bars represent measurements at study entry, and black bars represent measurements at the end of the study. Statistical analysis: ANCOVA with Bonferroni correction for between-group comparisons; paired *t* tests for within-group comparisons. * *p* < 0.05 compared with the corresponding values in group 1. ^#^ *p* < 0.05 compared with the corresponding values in group 3. ^&^ *p* < 0.05 compared with baseline values within the same group.

Under baseline conditions, the only difference in sexual functioning between the assessed groups was a higher score for desire and arousal in group 2 compared with the other two groups. In group 1, the effect of vitamin D on sexual functioning was limited to an increase in lubrication and lack of pain/discomfort scores. In group 2, an increase was observed in the total FSFI score as well as in scores for all assessed domains. In group 3, no effect of vitamin D supplementation on sexual functioning was observed. Group 2 differed from the remaining groups in terms of percentage changes in the total score and in scores for all domains, as well as in post-treatment values of all assessed FSFI parameters. In contrast, group 1 differed from group 3 with respect to the magnitude of changes in lubrication and lack of pain/discomfort scores in response to treatment, as well as the values of both domains on the final day of the study. At the conclusion of the study, group 2 also differed from the remaining groups in the proportion of patients with female sexual dysfunction (Figure 4 and Figure 5, Table 2).

### 2.5. Depressive Symptoms

At baseline, no statistically significant differences were observed between groups in total BDI-II scores or in the proportion of patients exhibiting depressive symptoms. Vitamin D treatment caused a reduction in both the total BDI-II score and the proportion of affected patients exclusively in group 2. Compared with the other groups, group 2 demonstrated significant differences in percentage changes in BDI-II scores, post-treatment BDI-II values, and the post-treatment proportion of patients with overall and mild depressive symptoms. The post-treatment BDI-II score was lower in group 1 than in group 3 (Figure 4, Table 2).

### 2.6. Correlations

At baseline, testosterone concentrations correlated with vitamin E intake, with positive correlations observed in groups 1 and 2 (r = 0.39, *p* < 0.01 and r = 0.41, *p* < 0.01, respectively), whereas a negative correlation was noted in group 3 (r = −0.27, *p* < 0.05). The effect of vitamin D on thyroid antibody titers correlated with their baseline levels (r = 0.53, *p* < 0.001 to r = 0.70, *p* < 0.001), with increases in 25OHD concentrations (r = 0.30, *p* < 0.05 to r = 0.49, *p* < 0.001), and with its effect on SPINA-GT values (r = 0.32, *p* < 0.05 to r = 0.44, *p* < 0.01). Positive correlations between the effect of vitamin D on thyroid antibody titers and on sexual function were observed in all study groups; in the case of desire and arousal, these correlations were also associated with its effect on testosterone levels. In group 3, correlations were additionally observed between changes in estradiol concentrations and effects on lubrication and lack of pain/discomfort scores (Table 3). The effect of vitamin D supplementation on the global FSFI score and on each of its domains correlated with vitamin E intake during the study, with positive correlations in groups 1 and 2 and negative correlations in group 3 (Table 4). Positive correlations were observed in all study groups between changes in BDI-II scores during treatment and the effect of vitamin D on overall sexual function and all of its domains (Table 5). No other correlations were identified.

## 3. Discussion

Before the initiation of vitamin D therapy, the studied patient groups differed exclusively with respect to testosterone concentration, which was highest in the group with adequate dietary vitamin E intake. Given the patient selection procedure, this finding is unlikely to reflect baseline differences in the severity of the autoimmune process. Similar baseline characteristics and the absence of correlations suggest that differences in testosterone concentrations were also unrelated to hypothalamic–pituitary–thyroid axis activity or vitamin D status. A potential explanation may involve differences in vitamin E intake, as supported by the nonlinear association between vitamin E consumption and serum testosterone concentrations. This interpretation is consistent with previous studies in women of reproductive age. Insufficient dietary vitamin E intake was associated with lower concentrations of sex hormone-binding globulin (SHBG), a major regulator of circulating total testosterone concentrations [30]. Conversely, high-dose vitamin E supplementation decreased total testosterone concentrations in women with polycystic metabolic ovarian syndrome, possibly due to reduced cholesterol availability, as cholesterol serves as a precursor for testosterone biosynthesis [31]. Testosterone plays a key role in regulating women’s sexual health, with its impact most pronounced on desire and arousal [32,33]. Thus, higher baseline levels of this hormone may account for the higher scores observed in both domains among patients with adequate vitamin E intake.

Considering the relationship between antithyroid antibody titers (particularly TPOAb) and the risk of progression from the euthyroid stage of Hashimoto’s disease to hypothyroidism [34], as well as other adverse consequences of this condition, including endothelial dysfunction, systemic inflammation, and damage to central nervous system cells [35,36,37], efforts should be directed toward achieving the greatest possible reduction in the activity of the autoimmune process in the thyroid gland. Thus, an interesting finding of the present study was the association between the beneficial effects of exogenous vitamin D on thyroid antibody titers and vitamin E intake. They seem to support the rationale for optimizing vitamin E intake and avoiding both inadequate intake of this vitamin and high-dose vitamin E supplementation, despite the excellent tolerability of high-dose supplementation throughout the entire study period.

Given that the primary endpoint of this study was the assessment of sexual functioning, the most significant finding was that the relationship between the effect of calciferol on sexual response and vitamin E intake in euthyroid women with Hashimoto’s disease receiving exogenous vitamin D supplementation follows an asymmetric curve. Improvements in overall sexual functioning and across all evaluated functional domains were observed only when vitamin E intake remained within the recommended range. This effect was independent of whether adequate vitamin E intake was achieved through dietary sources alone or through a combination of diet and low-dose vitamin E supplementation. Interestingly, the range of total daily vitamin E intake associated with the maximal beneficial effect on sexual function corresponds to the intake levels reported in previous studies by other investigators [27,28,29]. Since the effect of exogenous calciferol administration among women with low vitamin E intake was limited to only two domains—lubrication and lack of pain/discomfort associated with intercourse—and remained moderate even within these domains, women of reproductive age with Hashimoto’s thyroiditis receiving vitamin D supplementation should ensure that their vitamin D intake does not fall below the Recommended Dietary Allowance established by the Institute of Medicine [38]. Moreover, lack of improvement or unsatisfactory improvement in sexual response should prompt a more thorough assessment of vitamin E status and possible supplementation. An even less favorable effect appears to be associated with vitamin E supplementation exceeding 400 mg/day, a level typically resulting from the use of high-dose vitamin E preparations. Such excessive vitamin E intake completely eliminated the beneficial effects of vitamin D supplementation on all aspects of sexual health in women with autoimmune thyroiditis and should therefore be avoided in this population.

The bidirectional nature of the correlations between sexual functioning and vitamin E intake suggests a complex interaction between vitamins D and E in relation to sexual functioning. An indirect effect of calciferol is further supported by the absence of direct correlations between changes in individual aspects of sexual functioning and serum 25OHD concentrations. This effect does not appear to result from altered vitamin D absorption, metabolism, or excretion, as evidenced by comparable baseline and endpoint 25OHD concentrations (the former reflecting the group selection protocol). The most likely explanation for the between-group differences in sexual functioning is a reduction in thyroid autoimmunity, supported by three observations. First, the effect of vitamin D on TPOAb and TgAb titers was greatest in individuals with adequate vitamin E intake. Second, changes in both antibody titers correlated with increases in 25OHD concentrations and improvements in female sexual functioning, with the strongest correlations observed in the adequate vitamin E group. Third, TPOAb titers, considered more sensitive and specific for autoimmune thyroiditis than TgAb [34], decreased in women with low vitamin E intake but not with high vitamin E intake. Although the reduction in inflammatory activity in the adequate vitamin E group was accompanied by an increase in SPINA-GT, the absence of correlations between this parameter and FSFI domain scores argues against an association between improved sexual functioning and improved thyroid secretory function [39]. The stronger anti-inflammatory effect of vitamin D in the presence of adequate vitamin E intake may result from complementary molecular actions of these two vitamins. Through activation of VDR, vitamin D suppresses pro-inflammatory signaling pathways, including nuclear factor kappa-light-chain-enhancer of activated B cells (NF-κB) signaling [40,41], whereas vitamin E reduces oxidative stress and lipid peroxidation [42], thereby potentially enhancing the anti-inflammatory effects of vitamin D. Insufficient vitamin E intake may disrupt redox homeostasis and attenuate this interaction. Furthermore, VDR-mediated genomic signaling requires heterodimerization with RXR; therefore, alterations in RXR-related signaling associated with vitamin E deficiency could potentially influence VDR activity [21]. Redox-inflammatory interactions may also contribute to the limited effect of vitamin D supplementation in women with excessive vitamin E intake. Under such conditions, a shift from antioxidant to pro-oxidant activity may occur due to the accumulation of tocopheroxyl radicals. In the presence of specific factors, such as metabolic dysregulation, transition metals, or genetic susceptibility, these radicals may exert pro-oxidant effects and counteract the anti-inflammatory actions of vitamin D [43].

Among the assessed non-thyroid hormones, differences in sexual functioning among women with autoimmune thyroiditis receiving calciferol may be partly associated with changes in testosterone and estradiol levels. The increase in serum testosterone in response to vitamin D treatment was observed only in women with adequate vitamin E intake, which suggests a potential interaction between vitamins D and E in testosterone regulation. This finding is consistent with Chang et al. [44], who reported an association between serum 25OHD and testosterone concentrations in healthy reproductive-age women, with stronger associations observed with improved vitamin D status. Moreover, changes in the concentration of this hormone correlated with changes in desire and arousal, with the strength of these correlations being greater than that observed for antithyroid antibodies. This may suggest that alterations in testosterone levels play a more important role than modulation of the inflammatory process in mediating the effects of vitamins D and E on these two domains. VDR is expressed in the ovary and adrenal glands, and its activation by calcitriol may modulate the expression of steroidogenesis-related genes [45]. By reducing oxidative damage to steroidogenic enzymes, vitamin E may enhance steroid hormone biosynthesis [21]. Regarding estradiol, the inhibitory effect of vitamin D on its levels may explain the lack of improvement in lubrication and pain/discomfort domains among women with high vitamin E intake. Beyond the correlation analyses, this interpretation is supported by findings from previous studies. Adequate estrogen levels have been shown to be necessary for proper vaginal and vestibular lubrication and vaginal relaxation [46,47], whereas high doses of vitamin E have been shown to reduce estrogen production by inhibiting aromatase activity [48].

In addition to mechanisms discussed above, other pathways may contribute to the observed effects of vitamin D on women’s sexual health. The findings are unlikely to be explained by selenium homeostasis, as selenium status, assessed using the SPINA-GD index [39,49], did not differ between groups throughout the study period, suggesting that vitamin D supplementation did not affect selenium metabolism. A more plausible explanation involves vascular mechanisms, as both vitamins contribute to the regulation of blood flow, including within genital tissues [50]. Furthermore, both vitamin E deficiency [51] and excess [52,53] may impair this regulation. Potential interactions between the two vitamins at the level of neural transmission also warrant consideration, given their roles in maintaining proper innervation of target organs, which is essential for a normal sexual response [54,55]. Finally, despite the absence of differences in the distribution of potential confounding factors, residual confounding may still have influenced the findings.

The findings also suggest an interaction between the two vitamins in relation to their effects on mood. This observation is of particular importance in the context of the well-documented antidepressant effects of vitamin D in cases of mild mood disturbances [4,56], which are frequently observed in patients with autoimmune thyroiditis [57,58]. A significant reduction in depressive symptom severity and in the proportion of patients meeting the criteria for depression was observed only in the group with adequate vitamin E intake. Moreover, differences in sexual functioning between patients with low and high vitamin E intake at the end of the study were paralleled by a small but significant difference in BDI-II scores. The findings are more consistent with the interpretation that changes in mood are secondary to changes in sexual functioning than with the reverse relationship. Unlike sexual functioning, no correlations were observed between changes in BDI-II–assessed parameters and increases in serum 25OHD concentrations, changes in antithyroid antibody titers, measured hormone concentrations, or calculated parameters of thyroid homeostasis, regardless of vitamin E intake levels. In turn, the absence of baseline differences in BDI-II scores or in the proportion of patients meeting criteria for depression provides evidence against a direct role of vitamin E intake in modulating mood. Considering the moderate strength of the observed correlations, the impact on sexual response only partly explains the different effects on depressive symptoms. The lack of other intergroup differences or correlations with BDI-II scores prevents identification of nonsexual factors that may modulate the effects of exogenous calciferol on depressive symptoms. Potential contributors include systemic inflammation secondary to thyroid disease and interactions between vitamins D and E within central nervous system structures involved in emotional regulation, where both the vitamin D receptor and 1α-hydroxylase are expressed [59], and vitamin E may prevent deleterious lipid peroxidation [60].

Serum vitamin E levels were not measured due to the lack of access to liquid chromatography–tandem mass spectrometry, the reference method for accurate vitamin E quantification in clinical settings [61]. Although commercially available immunoenzymatic assays exist, their use is limited by insufficient specificity and the inability to reliably distinguish among tocopherol isoforms [61]. The evaluation of vitamin E homeostasis is further complicated by its accumulation in adipose tissue [51,62]. To improve the reliability of vitamin E intake assessment, dietary intake was evaluated on four occasions, including three assessments conducted during the vitamin D supplementation period, covering one-eighth of the supplementation period. Moreover, the exclusion criteria included comorbidities and medications that could affect vitamin E absorption and metabolism. However, interindividual variability in vitamin E pharmacokinetics cannot be excluded; therefore, the results should be interpreted with caution.

Additional limitations of the study protocol should also be acknowledged. Although the sample size exceeded that determined by power analysis, it remained relatively small, which may limit the extent to which the findings can be generalized to the broader population. Despite the validation of the FSFI and BDI-II questionnaires, potential limitations include misinterpretation of questions, recall bias, subjective responses, and the possibility that responses may not accurately reflect actual conditions. The cohort design of the study introduced risks such as selection bias, changes in disease severity over time, and the influence of uncontrolled variables as confounding factors [63]. While mandatory salt iodization in Poland ensured adequate iodine intake in the study population, selenium intake was insufficient [64,65], and its potential effect on the results cannot be excluded. The study included participants across a broad age range, which may have introduced confounding effects associated with age-related physiological changes. Finally, the possibility of false-positive results due to regression to the mean cannot be entirely excluded [66].

## 4. Materials and Methods

All procedures were conducted in accordance with the ethical principles outlined in the Declaration of Helsinki and its subsequent revisions. To ensure adherence to safety, ethical, and legal standards, the study protocol was reviewed and approved by the Institutional Review Board. All participants provided written informed consent after receiving a comprehensive explanation of the study’s objectives, significance, and potential risks and benefits.

### 4.1. Study Population

This prospective outpatient cohort study enrolled female participants aged 18–45 years with newly diagnosed euthyroid autoimmune thyroiditis and no prior history of treatment. Participants were required to meet the following inclusion criteria: (a) TPOAb titers exceeding 100 U/mL; (b) ultrasonographic features suggestive of autoimmune thyroiditis, including diffuse hypoechoic texture, heterogeneous parenchyma, hyperechogenic septations, and/or hypoechoic micronodules [67]; and (c) normal hypothalamic–pituitary–thyroid axis function, defined as serum thyroid-stimulating hormone (TSH) levels between 0.4 and 4.5 mIU/L, serum free thyroxine concentrations of 10.0–21.2 pmol/L, and serum free triiodothyronine concentrations of 2.2–6.7 pmol/L. To minimize the potential impact of seasonal variations in vitamin D metabolism and other laboratory and behavioral parameters under study, recruitment was structured to ensure that each study group included a comparable number of participants across all seasons [68]. Study participants were assigned to one of three groups according to total daily vitamin E intake: low (group 1), adequate (group 2), or high (group 3). The cutoff between low and adequate intake was defined as 15 mg (22.35 IU), corresponding to the recommended dietary allowance established by the Institute of Medicine [38]. The upper limit for adequate intake was arbitrarily established at 200 mg/day. Participants were assigned to group 3 if their vitamin E consumption exceeded 400 mg/day, a threshold chosen based on a meta-analysis by Miller et al. [69], which demonstrated that intake above this level is associated with an increased risk of all-cause mortality. For dosing in international units, one IU was considered equivalent to 0.67 mg for natural preparations and 0.45 mg for synthetic preparations. Individuals whose intake fell above the upper limit for group 2 but below the threshold for group 3 were excluded from the study. Vitamin E supplementation was permitted, provided that the supplements contained vitamin E as the sole biologically active ingredient and did not include other vitamins, antioxidants, or bioactive compounds. Intake from these supplements was included in the total vitamin E intake.

The target enrollment was set at 24 participants per study group, based on an a priori sample size calculation incorporating an anticipated allowance for potential participant dropout. The sample size calculation indicated that a total of 63 participants (21 women per group) would be required to detect the prespecified between-group difference in total FSFI scores, the primary study endpoint, with 80% statistical power and a two-sided α level of 0.05. The assumed standardized effect size (Cohen’s d = 0.5) was derived from findings of our previous study evaluating the effects of vitamin D, selenomethionine, and myo-inositol supplementation on female sexual function among reproductive-aged women with euthyroid Hashimoto’s thyroiditis [6]. To reduce baseline differences between the study groups, participants for groups 1 and 2 were selected from a larger pool of eligible candidates (Figure 1). Conversely, given the limited number of individuals with high vitamin E intake, all such participants were enrolled. This selection strategy aimed to ensure comparable mean values of age, TPOAb titers, and 25OHD levels across all study groups.

Participants were excluded if they tested positive for TSH receptor antibodies; had other endocrine or autoimmune disorders; cardiovascular, renal, or hepatic disease; gastrointestinal disorders associated with impaired digestion or absorption; neoplasms; anemia; abetalipoproteinemia or hypobetalipoproteinemia; premature ovarian insufficiency; alcohol or substance use disorders; or any other serious medical conditions. Additional exclusion criteria included pregnancy or lactation; dysmenorrhea; perimenopausal or postmenopausal status; congenital or acquired abnormalities of the reproductive system; a history of childhood sexual abuse or other traumatic experiences; a history of urogynecological surgery that could potentially affect sexual function; regularly sleeping fewer than 7 h per night; heavy smoking (at least 20 cigarettes per day); and current use of medications, including hormonal contraceptives, that could affect study outcomes.

### 4.2. Research Design

Over the six-month study period, participants received exogenous vitamin D at a daily dose of 100 µg (4000 IU), administered once daily in the morning. No adjustments to the dosage were permitted. For participants using vitamin E supplements, the existing supplementation regimen was maintained throughout the study period. They were allowed to use acetaminophen, nonsteroidal anti-inflammatory drugs, antibacterial and antiviral agents, cough suppressants, laxatives, antidiarrheals, or sleep-inducing medications, provided that treatment duration did not exceed 10 days and was discontinued at least eight weeks prior to study completion. Participants were also advised to maintain their habitual lifestyle, including meal frequency and portion sizes, dietary preferences, physical activity levels, and sleep duration.

Participants’ adherence was monitored every eight weeks by counting returned pills, with compliance defined as 90–110% of tablets or capsules consumed. They were also instructed to maintain their usual lifestyle habits throughout the study. Dietary intake of vitamins D and E (and other nutrients) was assessed using seven-day dietary logs. Their intakes were estimated by analyzing the types, composition, and portion sizes of consumed foods using authoritative food composition references [70]. This assessment was conducted at baseline and at two-month intervals during the vitamin D treatment period. Total daily vitamin D and vitamin E intakes were calculated by summing the amounts of vitamin D and vitamin E obtained from dietary sources and supplemental tablets or capsules. The resulting intake values for both vitamins during the period of exogenous vitamin D supplementation were averaged to determine the mean intake of these vitamins throughout the entire study period.

Serious adverse events were defined as any life-threatening occurrence, hospitalization or prolongation of an existing hospital stay exceeding 24 h, persistent or clinically significant disability or substantial interference with normal daily activities, events posing an immediate risk to the participant, or incidents requiring medical or surgical intervention to prevent such outcomes [71].

### 4.3. Laboratory Testing

Laboratory testing was conducted at the beginning of the study and repeated at the conclusion of vitamin D therapy. Venous blood samples were collected in the morning (07:30–08:30) following a 12-h fasting period. In all participants, sampling was performed during the early follicular phase of the menstrual cycle (days 2–5). Participants remained seated for at least 30 min prior to venipuncture to ensure physiological stabilization. Titers of TPOAb and TgAb, along with serum levels of 25OHD, TSH, free thyroid hormones, estradiol, testosterone, and prolactin, were measured using direct chemiluminescence with acridinium ester technology (ADVIA Centaur XP, Siemens Healthcare Diagnostics, Munich, Germany). All assays were performed in duplicate, and the mean of the two measurements was used to ensure accuracy and reliability. TSH, free thyroxine, and free triiodothyronine were then used to calculate thyroid homeostasis parameters, including Jostel’s TSH index and SPINA-derived indices for thyroid secretory capacity (SPINA-GT) and peripheral deiodinase activity (SPINA-GD) [39,49].

### 4.4. Questionnaire Analysis

Questionnaire responses were collected immediately after blood sampling, with both participants and investigators remaining blinded to the results of the biochemical tests throughout this period. Investigators were not permitted to influence the responses and were trained to refrain from providing personal opinions or guidance.

The initial questionnaire was designed to collect essential information regarding participants’ sociodemographic characteristics.

Sexual function was assessed using the FSFI, a 19-item instrument evaluating six distinct domains of female sexual function over the preceding four weeks: desire (2 items), arousal (4 items), lubrication (4 items), orgasm (3 items), satisfaction (3 items), and lack of pain/discomfort during penetration (3 items) [72]. Items are rated on a 5-point Likert scale ranging from 0–5 or 1–5 within each domain, with a “no sexual activity” option (scored as 0) available for four domains: arousal, lubrication, orgasm, and satisfaction [73]. To calculate the total FSFI score, each domain score is first multiplied by a domain-specific factor (0.6 for desire; 0.3 for arousal; 0.3 for lubrication; 0.4 for orgasm; 0.4 for satisfaction; and 0.4 for lack of pain/discomfort) to standardize domain totals on a comparable scale. The adjusted domain scores are then summed to obtain the overall FSFI score. Total scores range from 2 to 36, with higher scores indicating better sexual function [72]. A cut-off FSFI score of 26.55 was used to distinguish between women with and without sexual dysfunction [73].

The final questionnaire completed by participants was the BDI-II, a 21-item instrument assessing somatic, cognitive, and affective symptoms of depression over the preceding two weeks [74]. The BDI-II was developed to align with the depressive criteria outlined in the fourth edition of the Diagnostic and Statistical Manual of Mental Disorders (DSM-IV) [75]. Each item is scored on a scale from 0 to 3, with higher total scores reflecting greater severity of depressive symptoms. Scores are summed to yield the total BDI-II score, which can range from 0 to 63. A BDI-II score of 0–13 indicates a normal mood, 14–19 corresponds to mild depression, 20–28 reflects moderate depression, and 29–63 signifies severe depression [74].

### 4.5. Statistical Methods

Continuous variables were log-transformed to approximate normality. The study groups were compared using analysis of covariance (ANCOVA). The models were adjusted for smoking status, body mass index, and arterial pressure. When post-intervention values were compared, baseline measurements of the respective outcomes were included as additional covariates. Post hoc pairwise comparisons of adjusted means were performed using the Bonferroni correction for multiple comparisons. Within-group changes from baseline to post-intervention were assessed using paired *t* tests. Categorical variables were analyzed using the chi-square test. Associations between variables were assessed using Pearson’s correlation for continuous variables, point-biserial correlation for associations between binary categorical and continuous variables, and the phi coefficient for associations between two binary categorical variables. Statistical significance was defined as a two-tailed *p*-value < 0.05 after adjustment for multiple comparisons, as appropriate.

## 5. Conclusions

In women with Hashimoto’s disease who are euthyroid, both insufficient and excessive dietary vitamin E intake may contribute to diminished sexual desire and arousal. Administration of exogenous vitamin D appears to be most effective when vitamin E intake is maintained within a desirable range, with improvements observed across all FSFI-assessed domains. Low vitamin E intake appears to limit these benefits to lubrication and lack of pain/discomfort, whereas excessive intake may counteract them. Improvements in sexual function likely result from a reduction in thyroid autoimmunity and, in cases of adequate vitamin E intake, from an increase in testosterone levels. Positive effects on sexual health are also associated with mood improvement, which is observed only with sufficient vitamin E intake. These findings underscore the potential importance of ensuring adequate vitamin E intake during calciferol therapy for euthyroid autoimmune thyroiditis. However, although this pilot study yielded novel findings, the study protocol had inherent limitations; therefore, validation in multicenter, randomized clinical trials is warranted.

## Figures and Tables

**Table 1:** Baseline demographic, anthropometric, and laboratory parameters of the study cohorts (intention-to-treat analysis).

Variable	Group 1	Group 2	Group 3
Number of patients	24	24	24
Age (years)	34 ± 7	35 ± 7	34 ± 7
Primary or vocational/secondary/university education (%)	12/38/50	8/38/54	8/42/50
Employed/Blue-collar/white-collar/pink-collar workers (%)	96/12/38/46	96/16/38/42	92/21/29/42
Physical activity: total/several times a week/once a week/once a month (%)	87/50/25/12	96/54/29/12	92/54/29/8
Number of sexual partners (*n*)	2.0 ± 0.8	2.1 ± 0.8	2.2 ± 0.9
Number of marriages (*n*)/duration of marriages (months)	1.1 ± 0.5/86 ± 37	1.2 ± 0.6/90 ± 40	1.2 ± 0.5/83 ± 39
Number of deliveries (*n*)/Number of miscarriages (*n*)	1.2 ± 0.6/0.4 ± 0.2	1.3 ± 0.5/0.4 ± 0.2	1.3 ± 0.6/0.4 ± 0.2
Smokers (%)/Number of cigarettes a day (*n*)/Smoking duration (months)	38/7 ± 4/86 ± 53	42/6 ± 4/90 ± 61	42/6 ± 5/92 ± 57
Body mass index (kg/m^2^)	24.4 ± 4.7	23.4 ± 4.1	24.0 ± 4.1
Systolic blood pressure (mm Hg)	124 ± 22	122 ± 25	125 ± 22
Diastolic blood pressure (mm Hg)	76 ± 6	75 ± 7	77 ± 7
Mean dietary vitamin D intake (µg)	13 ± 8	14 ± 8	14 ± 8
Vitamin D deficiency/insufficiency (%)	50	50	46
Mean dietary vitamin E intake (mg)	8 ± 4	39 ± 43 *	808 ± 219 *^#^
Vitamin E supplement users (%)	0	42 *	100 *^#^
TPOAb (IU/mL)	898 ± 294	860 ± 312	884 ± 269
TgAb (IU/mL)	860 ± 395	848 ± 415	827 ± 388
TSH (mIU/L)	2.7 ± 1.4	2.9 ± 1.1	2.7 ± 1.3
Free thyroxine	13.4 ± 3.0	13.7 ± 2.5	14.1 ± 2.9
Free triiodothyronine (pmol/L)	3.1 ± 0.8	3.2 ± 0.8	3.2 ± 0.7
25OHD (ng/mL)	25.7 ± 13.9	26.5 ± 12.7	27.5 ± 11.8
Jostel’s TSH index	2.8 ± 0.2	2.9 ± 0.2	2.9 ± 0.3
SPINA-GT (pmol/s)	2.05 ± 0.46	2.03 ± 0.48	2.16 ± 0.50
SPINA-GD (nmol/s)	21.39 ± 4.85	21.60 ± 5.28	20.98 ± 5.10
Estradiol (pmol/L)	272 ± 122	298 ± 110	314 ± 97
Testosterone (nmol/L)	0.82 ± 0.38	1.19 ± 0.37 *^&^	0.86 ± 0.35
Prolactin (ng/mL)	15.4 ± 6.9	14.9 ± 7.4	14.3 ± 7.9
FSFI	25.35 ± 4.27	26.75 ± 4.19	25.35 ± 4.62
BDI-II	14.6 ± 4.0	14.0 ± 4.2	15.1 ± 4.4

Group 1 had low vitamin E intake, group 2 had adequate vitamin E intake, and group 3 had high vitamin E intake. Values are shown as the mean ± standard deviation, unless indicated otherwise. * *p* < 0.05 vs. group 1; ^#^ *p* < 0.05 vs. group 2; ^&^ vs. group 3.

**Table 2:** Sexual dysfunction and depressive symptoms of varying severity in reproductive-age women with euthyroid autoimmune thyroiditis before and after vitamin D treatment, stratified by vitamin E status.

Variable	Group 1	Group 2	Group 3
**FSFI score ≤ 26.55** (n [%])			
At baseline	14 [61]	13 [54]	14 [61]
At study completion	12 [52]	3 [12] *^#^	13 [56]
**Depressive symptoms** (n [%])			
At baseline	13 [56]	14 [58]	13 [56]
At study completion	10 [44]	6 [25] *^#^	13 [56]
**Mild symptoms** (n [%])			
At baseline	12 [50]	13 [54]	11 [48]
At study completion	10 [44]	6 [25] *^#^	12 [52]
**Moderate symptoms** (n [%])			
At baseline	1 [4]	1 [4]	2 [8]
At study completion	0 [0]	0 [0]	1 [4]
**Severe symptoms** (n [%])			
At baseline	0 [0]	0 [0]	0 [0]
At study completion	0 [0]	0 [0]	0 [0]

Group 1 had low vitamin E intake, group 2 had adequate vitamin E intake, and group 3 had high vitamin E intake. * *p* < 0.05 compared with the corresponding values in groups 1 and 3. ^#^ *p* < 0.05 compared with baseline values within the same group.

**Table 3:** Relationships between the impact of exogenous vitamin D on biochemical markers and sexual function in the study cohorts.

Correlated Variables		Group 1	Group 2	Group 3
Δ FSFI score	Δ TPOAb	0.40 ^b^	0.47 ^c^	0.35 ^2^
Δ FSFI score	Δ TgAb	0.38 ^b^	0.49 ^c^	0.31 ^a^
Δ Desire	Δ TPOAb	0.29 ^a^	0.35 ^b^	0.29 ^a^
Δ Desire	Δ TgAb	0.26 ^a^	0.36 ^b^	0.29 ^a^
Δ Desire	Δ Testosterone	0.45 ^b^	0.52 ^b^	0.46 ^b^
Δ Arousal	Δ TPOAb	0.31 ^b^	0.38 ^b^	0.28 ^a^
Δ Arousal	Δ TgAb	0.25 ^a^	0.35 ^b^	0.30 ^a^
Δ Arousal	Δ Testosterone	0.42 ^b^	0.51 ^c^	0.39 ^b^
Δ Lubrication	Δ TPOAb	0.40 ^b^	0.48 ^c^	0.41 ^b^
Δ Lubrication	Δ TgAb	0.32 ^b^	0.40 ^b^	0.29 ^a^
Δ Lubrication	Δ Estradiol	0.05	0.11	0.37 ^b^
Δ Orgasm	Δ TPOAb	0.35 ^b^	0.47 ^c^	0.28 ^a^
Δ Orgasm	Δ TgAb	0.31 ^a^	0.43 ^b^	0.27 ^a^
Δ Sexual satisfaction	Δ TPOAb	0.47 ^c^	0.42 ^b^	0.35 ^b^
Δ Sexual satisfaction	Δ TgAb	0.36 ^b^	0.42 ^b^	0.29 ^a^
Δ Lack of pain/discomfort	Δ TPOAb	0.35 ^b^	0.43 ^b^	0.38 ^b^
Δ Lack of pain/discomfort	Δ TgAb	0.32 ^a^	0.41 ^b^	0.27 ^a^
Δ Lack of pain/discomfort	Δ Estradiol	0.07	0.12	0.39 ^b^

Group 1 had low vitamin E intake, group 2 had adequate vitamin E intake, and group 3 had high vitamin E intake. ^a^ *p* < 0.05, ^b^ *p* < 0.01, ^c^ *p* < 0.001.

**Table 4:** Relationships between the impact of exogenous vitamin D on sexual function and mean daily vitamin E intake during the study in the study cohorts.

Correlated Variable	Group 1	Group 2	Group 3
Δ FSFI score	0.42 ^b^	0.46 ^c^	−0.34 ^a^
Δ Desire	0.38 ^b^	0.53 ^c^	−0.39 ^b^
Δ Arousal	0.43 ^b^	0.47 ^c^	−0.28 ^a^
Δ Lubrication	0.39 ^b^	0.42 ^b^	−0.42 ^b^
Δ Orgasm	0.29 ^a^	0.38 ^b^	−0.29 ^a^
Δ Sexual satisfaction	0.48 ^c^	0.52 ^c^	−0.28 ^a^
Δ Lack of pain/discomfort	0.41 ^b^	0.46 ^b^	−0.41 ^b^

Group 1 had low vitamin E intake, group 2 had adequate vitamin E intake, and group 3 had high vitamin E intake. Values are reported as correlation coefficients (r). ^a^ *p* < 0.05, ^b^ *p* < 0.01, ^c^ *p* < 0.001.

**Table 5:** Relationships between the impact of exogenous vitamin D on sexual function and depressive symptoms in the study cohorts.

Correlated Variables		Group 1	Group 2	Group 3
Δ BDI-II	Δ FSFI score	0.46 ^b^	0.51 ^c^	0.37 ^b^
Δ BDI-II	Δ Desire	0.44 ^b^	0.49 ^c^	0.42 ^b^
Δ BDI-II	Δ Arousal	0.39 ^b^	0.52 ^c^	0.38 ^b^
Δ BDI-II	Δ Lubrication	0.36 ^a^	0.46 ^b^	0.32 ^a^
Δ BDI-II	Δ Orgasm	0.38 ^b^	0.42 ^b^	0.29 ^a^
Δ BDI-II	Δ Sexual satisfaction	0.41 ^b^	0.53 ^c^	0.39 ^b^
Δ BDI-II	Δ Lack of pain/discomfort	0.35 ^a^	0.46 ^b^	0.28 ^a^

Group 1 had low vitamin E intake, group 2 had adequate vitamin E intake, and group 3 had high vitamin E intake. Values are reported as correlation coefficients (r). ^a^ *p* < 0.05, ^b^ *p* < 0.01, ^c^ *p* < 0.001.

## Data Availability

The data that support the findings of this study are available from the corresponding author upon reasonable request.
